# Cholestatic hepatitis in acute Epstein–Barr virus infection: A case report

**DOI:** 10.1002/ccr3.9357

**Published:** 2024-08-21

**Authors:** Amey Joshi, Divij Jha, Eki Wari, Moiz Saeed, Murtaza Hussain, Tadd Kaeo Hiatt

**Affiliations:** ^1^ Department of Internal Medicine Sparrow Hospital—Michigan State University East Lansing Michigan USA; ^2^ Department of Gastroenterology Sparrow Hospital‐University of Michigan East Lansing Michigan USA

**Keywords:** cholestasis, Epstein–Barr virus, hepatitis, transaminitis, viral infection cholestasis

## Abstract

Cholestatic hepatitis is a rare complication of Epstein–Barr virus infection and is often self‐limiting. Systemic signs, including viral prodrome and lymphadenopathy, can provide crucial diagnostic cues and avoid unnecessary invasive investigations or aggressive medical therapy unless indicated.

## 1 INTRODUCTION

Epstein–Barr virus (EBV) is prevalent in the general population of the United States, with approximately 90%–95% of adults exhibiting signs of previous EBV exposure by age 20.^1^ Infection with EBV typically manifests as infectious mononucleosis (IM), characterized by symptoms such as fever, lymphadenopathy, and pharyngitis. Temporary elevations in hepatic transaminases are frequently observed during the primary infection, but notable jaundice and cholestasis are uncommon, occurring in fewer than 5% of cases.^2^ Only a few cases of cholestatic hepatitis associated with EBV infection have been reported in the literature.^3^, ^4^, ^5^ We present a case of infectious mononucleosis complicated by cholestatic hepatitis that improved with supportive management.

## 2 CASE REPORT

A 20‐year‐old female with no medical or surgical history presented with complaints of fatigue, nausea, subjective fever and chills, myalgia, and throbbing right upper quadrant abdominal pain for 6 days duration. She denied any history of diarrhea, hematochezia, changes in menstrual cycles, insect or tick bites, or illicit drug use. She did endorse camping in Upper Michigan recently and occasional alcohol consumption without any reported binge episodes. On admission, she was noted to be febrile (101.3 F), tachycardic (110 beats/min), normotensive (108/65 mHg), and jaundiced. Physical examination revealed tender cervical adenopathy and minimal tenderness in the epigastric and right upper quadrant with notable hepatomegaly.

## 3 METHODS

Laboratory investigations were significant for lymphocytic‐predominant leukocytosis (12500/mm^3^) with an absolute lymphocyte count of 7.75 × 10^3^ /uL, elevated aspartate aminotransferase (AST) (310 IU/L), alanine aminotransferase (ALT) (284 IU/L), alkaline phosphatase (254 IU/L), and elevated total and direct bilirubin (4.4 and 3.5 mg/dL, respectively), and mildly elevated C‐reactive protein (1.7 mg/dL). Abdominal ultrasound was suggestive of a moderately thickened gall bladder with no evidence of cholelithiasis and a common bile duct that measured 3 mm. The ultrasound also revealed a centrilobular “starry sky” echogenicity of the liver suggestive of hepatitis. Magnetic resonance cholangiopancreatography revealed no biliary obstruction, dilation, or evidence of stones. MRI did reveal hepatomegaly of 20 cm, and splenomegaly of 17 cm. EBV viral capsid IgG and IgM were positive, indicating an acute infection. Further workup revealed negative HIV, Hepatitis B and C, toxoplasma, and cytomegalovirus (CMV) antibodies.

## 4 RESULTS

She was continued on supportive treatment with analgesics and fluid resuscitation. Through the course of her hospital stay over 4 days, the patient reported significant symptom relief with down‐trending of liver enzymes and bilirubin.

## 5 CONCLUSION

The present case highlights a rare presentation of EBV‐induced cholestasis. The classical history and physical exam findings in our case of malaise, low grade fevers, splenomegaly, and cervical lymphadenopathy were diagnostic cues leading to a higher suspicion of EBV‐related cholestasis. These findings may help providers narrow their diagnosis and avoid incessant and invasive testing. Supportive therapy often leads to resolution of symptoms and transaminitis and steroid therapy should be preserved for the complications associated with EBV infections including fulminant hepatitis or compromised airway.

## 6 DISCUSSION

EBV infection frequently involves the liver, manifesting as a transient elevation in liver enzymes in about 75% of the cases.^6^ Although cases of significant icterus and cholestasis have been documented, with an incidence of less than 5%, isolated EBV‐associated cholestasis is rare, with only a handful of cases being described.^3^

The pathophysiology underlying EBV‐associated cholestasis remains unclear. It is thought to be primarily due to an intense immune‐mediated rather than cytotoxic response.^7^ It has been hypothesized that EBV‐induced lymphocytic infiltration of the liver and proliferation of Kupffer cells results in mild intrahepatic and bile duct inflammation leading to cholestasis.^7^, ^8^ Other viruses, including CMV, hepatitis, and HIV, may also present with cholestatic jaundice, and these infections should also be screened in the setting of cholestasis in the absence of biliary dilation. Medications including erythromycin, clavulanic acid, estrogen, phenothiazines, and non‐steroidal anti‐inflammatory drugs have also been noted to be associated with cholestatic jaundice, and a thorough history should be obtained to rule out this possibility.^3^

Due to the wide range of potential differential diagnoses, a systematic approach should be used in investigating and managing a patient presenting with cholestasis. A detailed history, including recent travel, exposure to sick contacts, alcohol use, and medication, may help narrow the diagnosis. In viral pathologies, signs including hepatosplenomegaly, lymphadenopathy, and preceding viral prodrome may help in providing diagnostic cues.^9^, ^10^ In the setting of EBV‐induced cholestasis, laboratory investigations may be significant for lymphocytosis, as seen in the present case. Alanine transaminitis levels in EBV‐induced liver damage rarely exceed 1000 IU/L.^9^ Serology, including testing for capsid antigens, is crucial for making the diagnosis. Other potential causes of cholestasis must be ruled out, including obstructive etiologies and Magnetic resonance cholangiopancreatography (MRCP) is the diagnostic modality of choice.^11^

The treatment of EBV‐induced cholestasis is primarily supportive, as there are no specific antiviral medications for EBV.^10^ There is limited evidence of the use of medications such as acyclovir, ganciclovir, or famciclovir in managing these patients.^12^ Few studies have shown steroid administration to decrease the time of viral shedding. Steroid administration, however, was not found to decrease the time to return to work/school and hence is not routinely recommended.^13^, ^14^ In cases complicated by fulminant hepatitis or compromised airway, patients may be treated with high‐dose steroids to diminish the inflammatory response to the virus.^12^ Although most cases of EBV infection are self‐limiting, physicians need to be watchful for potential complications, including chronic active EBV infection, chronic active hepatitis, granulomatous hepatitis, vanishing bile duct syndrome, prolonged cholestasis, or acute liver failure may develop.^15^

## AUTHOR CONTRIBUTIONS

**Amey Joshi:** Conceptualization; data curation; formal analysis; investigation; methodology; project administration; writing – original draft; writing – review and editing. **Divij Jha:** Conceptualization; investigation; methodology; supervision; visualization; writing – original draft; writing – review and editing. **Eki Wari:** Conceptualization; data curation; project administration; resources; supervision; writing – original draft; writing – review and editing. **Moiz Saeed:** Conceptualization; investigation; methodology; project administration; supervision; writing – original draft; writing – review and editing. **Murtaza Hussain:** Conceptualization; project administration; resources; supervision; validation; visualization; writing – original draft; writing – review and editing. **Tadd Kaeo Hiatt:** Conceptualization; methodology; project administration; supervision; visualization; writing – original draft; writing – review and editing.

## CONFLICT OF INTEREST STATEMENT

The authors declare no conflicts of interest.

## CONSENT

Written informed consent was obtained from the patient to publish this report.

## Data Availability

The data supporting the findings of the present study are available from corresponding author upon request.
